# A framework to amplify the voices of underrepresented children and young people in rare disease research

**DOI:** 10.1186/s41687-026-01056-7

**Published:** 2026-03-31

**Authors:** L. Thomas, J. Preston, H. Hardwick, J.D. Peipert, L. Oni

**Affiliations:** 1 Department of Women’s and Children’s Health, University of Liverpool, Liverpool, UK; 2 Centre for Patient Reported Outcomes Research, Department of Applied Health Sciences, University of Birmingham, Birmingham, UK; 3 Department of Renal Medicine, UCL Centre for Kidney and Bladder Health, University College London, London, UK; 4 Department of Paediatric Nephrology, Great Ormond Street Hospital for Children NHS Trust, London, UK; 5 Paediatric Nephrology and Honorary Consultant Paediatric Nephrologist, Institute in the Park Building, Alder Hey Children’s Hospital, University of Liverpool, Eaton Road, Liverpool, L12 2AP UK

## Abstract

Rare diseases predominantly affect children and young people (CYP). Current research involving CYP often fails to adequately represent those from lower socioeconomic backgrounds, contributing to widening health and research inequalities. This concise report proposes a framework to build on successful methods that were able to engage with underrepresented CYP. This was achieved through partnership with social enterprise organisations and utilised connections in the community to provide positive knowledge exchange of local services that may enhance their socioeconomic situation, in turn for engaging on how to improve services or advance research. These insights, together with information from scoping the field, were used to develop a practical framework for inclusive Patient and Public Involvement and Engagement (PPIE) with CYP. This framework has been initially designed to be evaluated in CYP with rare kidney diseases, as an exemplar long term health condition. The model of ‘PPIE through collaborative empowerment’ leverages existing community trust to reduce barriers to participation, hopefully opening the door to more representative views from children residing in regions of low socioeconomic status. The framework has potential to be adapted to address other inequalities or adaptation for different diseases, hopefully offering a replicable approach to representative research design. The next stages of this research are to implement the framework and formally evaluate its success.

## Introduction

Over 7,000 rare diseases have been identified worldwide, and they disproportionately affect children and young people (CYP), resulting in long-term health conditions (LTHC) throughout the life course [1]. Unfortunately, over 30% of children with rare diseases will die before their fifth birthday, in part due to a lack of approved treatments appropriate for use within the paediatric population [2], indicating the urgent need to advance the translational pathway for patients living with rare diseases. Involvement of patients and the public is increasingly recognised as an essential component when planning clinical services or research, and this is termed patient and public involvement and engagement (PPIE). More specifically, PPIE is defined as ‘research being carried out with or by members of the public rather than to, about or for them’ [3]. Patients living with rare diseases require tailored approaches to management, especially as they mostly affect CYP who are considered a vulnerable group within society, and this extends across both medical management and research. If these are not designed appropriately, it can contribute to a culture of underrepresentation. Positive efforts to design services or research to meet the unique needs of CYP have been witnessed over the past few years with more frequent use of age-appropriate PPIE approaches. The challenge now is to ensure that these efforts are reflective of the entire population who experience the disease, and this requires overcoming the multi-factorial barriers to support inclusion.

Chronic kidney disease (CKD) is an example of a LTHC where advances to slow the progression to the end point of kidney failure are just emerging [4]. It progresses to an end point of kidney failure which has a devastating impact on the patient and their family. In the United Kingdom, research investigating the factors influencing the progression of kidney failure in patients with rare kidney diseases have identified that children and young people are significantly more likely to live in a deprived area when compared to adults living with rare kidney diseases [5, 6]. Whilst it is speculated that this may be related to the additional economic burden of caring responsibilities for children, it remains unknown whether the rare kidney disease contributed to a socioeconomic change, or the socioeconomic environment has been a trigger aggravating the rare kidney disease onset, and emphases the need to develop inclusive PPIE methods to understand the true needs of this population.

Whilst other barriers may also be important, such as ethnic status and neurodevelopmental ability, this concise report will focus firstly on the key factors informing the need for improvements, namely the rights of all children to be heard, addressing health inequalities, children with long term health conditions, and implementing PPIE into research design, and on methods to involve vulnerable CYP living with LTHC who reside in deprived areas, to address the specific barrier of socioeconomic deprivation through novel PPIE approaches. By focusing on developing a method of ‘PPIE through collaborative empowerment’, it aims to promote greater inclusion of CYP from underrepresented socioeconomic groups.

## Key factors influencing the need for inclusive PPIE

### Capturing representative inclusion to maintain children’s legal right to be heard

The culture of excluding children in service or research design conflicts with their legal rights as outlined in the 1989 United Nations (UN) convention on the rights of child which state that children have the right to be heard in all matters affecting them. Article 24 also highlights that there is a need to develop primary and preventative healthcare as children have the right to be healthy and to access appropriate treatment [7, 8]. In view of this legal right, listening to and acting upon the voices of CYP to design age-appropriate medical advances would allow advances in LTHC that are tailored to meet their needs. Failing to include children in the design of interventions carries the risk of services or treatments that may not be accessible or meet their needs appropriately, and go against their human rights to be heard, exacerbating exclusion. Meaningful PPIE practices help ensure that interactions with patients and families are productive to accurately capture their lived experience starting at an early stage in the research design and carried throughout the execution of any study, thereby ensuring that CYP remain the centre focus [9].

### Capturing representative inclusion to address health inequalities

Health inequalities are a persistent feature in research and are defined as ‘health disparities experienced within and between patient groups that are judged to be unfair, unjust, unavoidable and unnecessary’, and these are often stemming from underlying social, political, and economic structures [10]. Health inequalities can be reflected in suboptimal health outcomes, such as low life expectancies, within certain populations due to influences beyond their direct control. CYP experience greater health inequalities due to the added vulnerability of being a minor. Their wellbeing is shaped by a range of external factors, including government policies, circumstances of their caregivers, and the socioeconomic environment that they are born into. Notably the implementation of austerity measures that have been instigated across the United Kingdom, for example, have been linked to a rise in child poverty with anticipated long lasting negative implications, this can already be observed in the increasing infant mortality rate [11].

### Capturing representative inclusion for children with rare long-term health conditions (LTHC)

The definition of long-term health conditions can vary however they are typically defined as conditions which at present cannot be cured [12]. These conditions affect every aspect of a patient’s life, often persisting across the life course with long term reliance on health care services. CKD is a life-long condition that leads to the end point of kidney failure, after which kidney function must be replaced with dialysis machines or kidney transplants that have a limited lifespan. CKD in childhood adds another layer of vulnerability, affecting every aspect of a child’s life and reverberating through family dynamics and the lives of their caregivers. A study of 402 children living with CKD demonstrated significantly lower health-related quality of life in comparison to their peers from the general population, with impairments across physical, school, emotional, and social health domains [13] and an observational cohort of 29 children and their primary caregivers showed statistically significantly increased incidence of depression and anxiety in both the child and carer [14]. Children with CKD often require lifelong care across both paediatric and adult services and face a high economic burden due to increased care needs, which can limit employment and complicate transport to appointments [3]. In the UK, children with rare kidney diseases are more likely than adults with rare kidney diseases to live in areas of higher deprivation, as documented in the evaluation of 27,285 participants contributing to the National Registry of Rare Kidney Diseases [5, 7]. Racially marginalised CYP with physical disabilities face additional barriers, including isolation and language challenges, which can hinder access to care and research. These issues are often intensified if there are additional parental communication difficulties when navigating healthcare systems [14]. For children with kidney disease, the added complexity of evaluating the pharmacology of drugs through the differing stages of CKD provides further exclusion [15]. New research aiming to deliver advanced treatments for patients at risk, or experiencing, CKD are emerging, therefore ensuring representative recruitment, particularly among CYP in deprived areas, is essential to reach the target population experiencing disease [16].

### Additional barriers to implementing inclusive PPIE into clinical service or research design

Including CYP in research is essential to produce relevant and impactful healthcare studies within this population. However, conducting meaningful PPIE presents challenges, such as the need to balance the inconvenience to the child, and the specialist skills required for appropriate communication. Further barriers exist for colleagues working in pharmaceutical industry settings, where formal regulations may prevent direct access to minors for engagement purposes [17]. Whilst there are some outstanding initiatives in paediatric clinical research, namely the creation of Young People’s Advisory Groups (YPAGs), that are now commonplace in many international countries where they serve as contact points for clinicians, there remains a notable gap in PPIE that can capture the voice of underrepresented CYP. Currently, engagement is typically conducted at in-person events or using online meetings, which assumes the ability to travel or the ability to have access to suitable digital devices with a stable internet connection. CYP coming from low socio-economic backgrounds may not have the privilege to connect to PPIE through these routes, nor may improving health be their primary priority when socioeconomic circumstances mean their basic needs are not able to be met [18]. Previous research has been performed to establish a ‘minimum digital living standard (MDLS)’ for children and their families, meaning a minimum expectation that would allow digital engagement with the current era, although aimed at families without any additional health needs; a national study reported that 45% of households fell below this pre-defined threshold and additionally the caregivers frequently lacked the skills to digitally engage effectively [19]. Moreover, although YPAGs are designed for CYP, initial contact and consent occurs through parents or guardians. Therefore, if the caregiver lacks awareness, time and/or resources to engage, then opportunities for the CYP can be missed. Previous studies have shown that linking underrepresented patients, such as those living in deprived regions, with pre-established social enterprise initiatives who have the skills and knowledge to provide appropriate support to meet their basic needs first, such as access to parental mental health support, food banks or public finances to cover the cost of living expenses, provides a novel method to increase engagement and allows the acquisition of valuable feedback from CYP living with LTHC and socioeconomic disadvantage who may not otherwise be heard [20].

## A proposed model of PPIE to overcome socioeconomic disadvantage

### PPIE through a model of collaborative empowerment

**Fig. 1:**
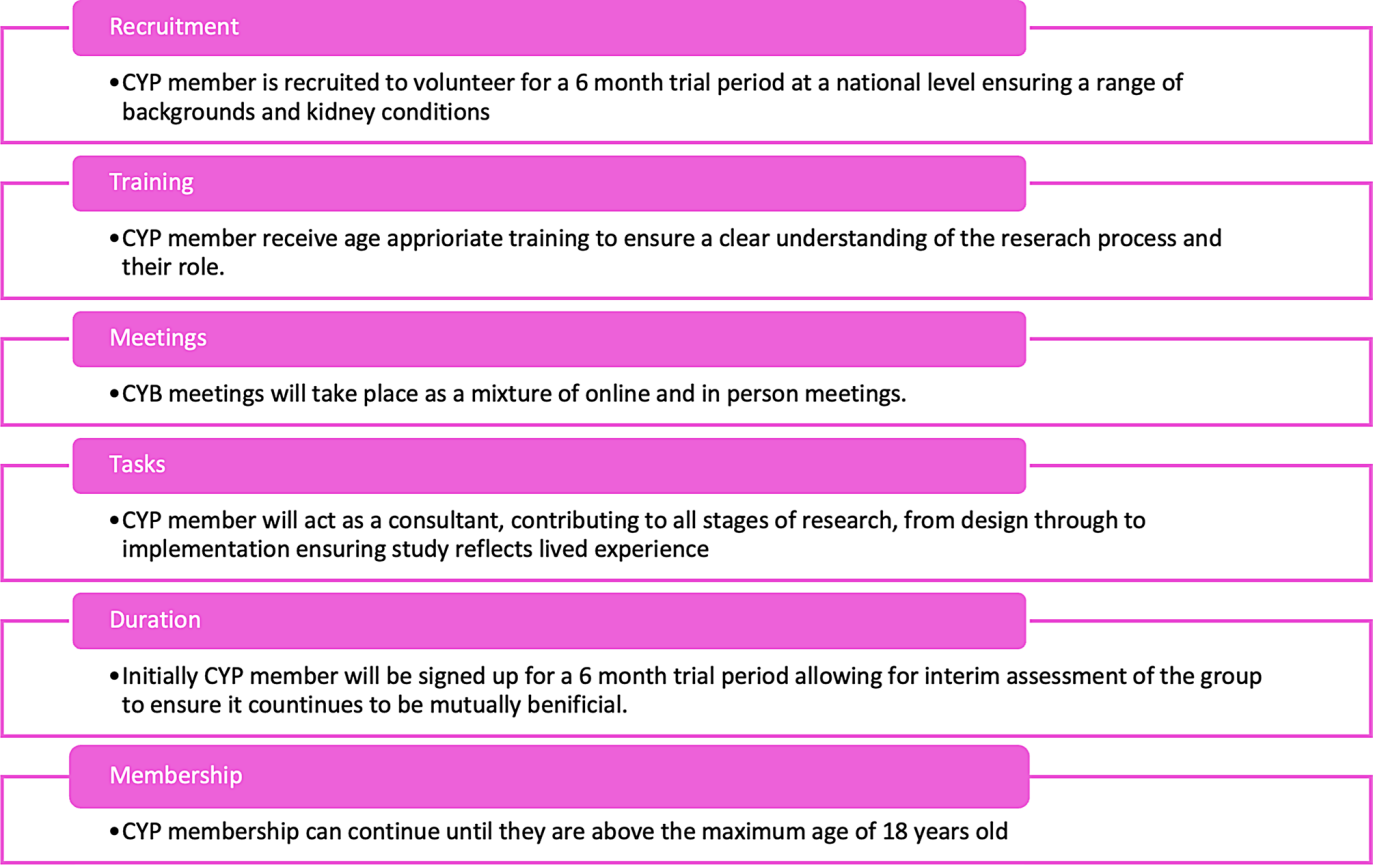
Process of CYP participation for rare kidney disease children and young Persons Advisory board

Connecting this prior work to achieve more meaningful involvement of CYP in research demands a structure that actively seeks, listens, and adapts to their needs of CYP from marginalised backgrounds. While the “Lundy Model of Participation” [21] offers an excellent framework for engagement, there remains limited guidance on effectively engaging underrepresented groups. It is already common practice to cover the financial aspects of PPIE and therefore this is assumed as a prior standard. Using kidney disease as a model, scoping of the existing methods used for PPIE were established and a multi-purpose ‘Rare Kidney Disease Children and Young Persons Advisory Group (R-KID YPAG) has been developed for children aged 8–18 years old with lived experience of rare kidney disease, with the aim of maximising inclusion for representative input into clinical research. The process of CYP participation in outlined in Fig. 1.

**Fig. 2:**
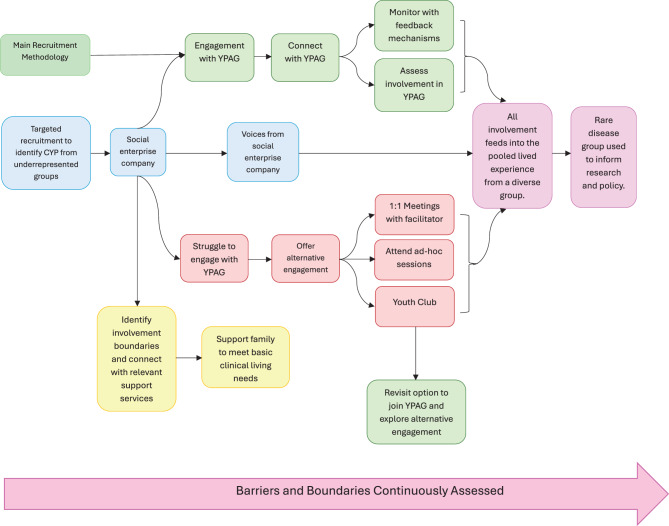
A framework for socio-economic inclusive PPIE using collaborative empowerment. The method uses pooled information collected through a collaborative approach outlined in this framework that will be used to ensure voices of CYP living in socioeconomic disadvantage are represented. The model uses direct contact, targeting populations, and gathering indirect information using a variety of PPIE approaches. The method is perpetually reassessed throughout the framework to continue to strive for inclusion and a representative pooled experience

The method utilises the group collaborating with social enterprise initiatives who can act as a safe intermediate to strengthen relationships with vulnerable populations to promote fair, more representative involvement. In amplifying the voices of those previously unheard CYP, this initiative aims to bridge socioeconomic gaps in research and facilitate a culture of inclusive practices. A structured mechanism will link social enterprise organisations, such as those located in the community who have access to relevant support for the entire familiy, to the R-KID YPAG with direct or indirect involvement of CYP from a range of socioeconomic circumstances using mixed methods to support perpetual engagement (Fig. 2). Perpetual review will help to identify underrepresented CYP, assess participation barriers, and keep exploring alternative routes for active participation. It will be re-evaluated on 6-monthly cycles to determine whether it has gained appropriate inclusion to permit parental consent and the child’s assent to participate, using a suitable period of engagement that has been agreed by young people and those with experience of managing PPIE groups. This collaborative empowerment model ensures that CYP, regardless of socioeconomic status, can contribute to inform research and policy. A parallel project will define the minimal digital living standard for children with kidney disease to provide a method to identify patients at risk of digital exclusion, which includes access to devices and data, ensuring there are adequate digital skills, and handling beliefs and trust in the digital era. It is anticipated that this work would further strengthen an inclusive model, which would then act as an enabler to connect children with charities committed to supporting the digital divide (eg: The Good Things Foundation, UK).

The inclusive CYP specialist group will be able to engage in several roles and responsibilities as shown in Table 1. An example of the utility of the proposed framework could be to enhance the design of patient reported outcome and experience measures (PROMs/PREMs). PROMs are developed using various tools to capture a patient’s experience of medical interventions or treatment directly without interpretations from clinicians or caregivers [22]. These measures hold a significant potential to empower patients by offering a standardised, scientifically rigorous way to document their priorities and care experiences. Critically, PROMs are increasingly encouraged by regulators to support evaluation of new treatments [23]. However, for PROMs/PREMs to truly reflect the realities of those affected, they must be accessible to all relevant groups. Recent literature has highlighted the importance of equitable access to PROMs, noting several barriers that particularly effect vulnerable populations [24, 25]. This includes the need to appropriately involve children who are capable of self- reporting (aged 8+) and methods to capture the views of younger children as appropriate.

**Table 1:** Proposed roles and responsibilities that the rare disease young person advisory group (YPAG) members could support to advance service development or research initiatives

Type of Involvement	Involvement Details
Advisory group	Researchers working on a trial or project can address the group to gain opinions and information to be used for research development.
Ad-Hoc events	Conferences, talks, and other educational or industry events. Attendance can be used to educate others or to provide education to members of the YPAG.
Group events	Group events targeted at improving the partnerships within the members. This may include tailored education on how they can support clinical research to ensure the YPAG members are continually expanding their own knowledge and securing the working relationships within the group.
Youth clubs	YPAG members can inform the formation and role of youth clubs in the community to enrich lives with the sole purpose of bonding and sharing lived experience.
1:1 engagement	CYP who prefer not to be involved in wider group events, may be better accommodated with smaller 1:1 in person or virtual meetings to gauge their views. To increase accessibility these meetings could align opportunistically with medical appointments.
Development of patient reported outcome measures	YPAG members can be used to inform the methods or engage directly in the development of patient-related materials, signal priorities for treatment and care, that in turn can lead to formal ways to develop patient reported outcome measures that meet the needs of this population.
Aiding funders in grant decision making	YPAG members can become experts by experience to participate as a valued member of grant boards to provide their views on funding allocation.

Existing PROMs that have been developed without input from marginalised groups, and therefore may require adaptation to meet their needs, otherwise they risk reinforcing barriers and health inequalities once implemented [23, 26]. For example, PROMs that have been designed by highly educated members of the public would require high levels of health literacy or reading comprehension for engagement which may be difficult for some patients to complete [27]. To ensure PROMs are inclusive, steps must be taken to align the content, wording, delivery schedules and modes of assessment with the diverse needs and resources of patient populations [25, 28]. Enhancing accessibility to the content of patient facing material can be achieved through online readability tools, and this remains an area that requires active improvement, as shown in evaluation of 109 paediatric patient information leaflets that demonstrated 81% were pitched at a ‘difficult’ reading level. [29] Meaningful inclusion of parents, with a focus on CYP and their families, in the design and clinical implementation of PROMs and patient facing materials, is essential to maximise participation and data quality. Further research is needed to understand how equity in PROM participation results in benefit to children. Following implementation of the proposed framework for rare kidney diseases, in a single region that has established social enterprise connections, an evaluation will be performed to determine whether it more accurately captures the voice of CYP to design service or research initiatives when compared to existing methods.

## Conclusion

As clinical medicine advances, it remains vital to address the barriers that continue to disadvantage many vulnerable CYP, including socioeconomic factors that are beyond their control. Whilst other factors influencing outcomes may also deserve attention, such as engagement with ethnic diverse groups and children with neurodisability, this framework specifically aims to capture the views of children residing in lower socioeconomic regions in the first instance. Given the complexity and low prevalence of rare disease, inclusive tailored research methodologies are essential to overcome participation barriers and capture accurate findings. The next stage of this research is to implement and review the proposed framework for inclusive PPIE to ensure that it achieves this goal.

## Data Availability

No raw data was involved in this manuscript however any information will be made available upon reasonable request to the corresponding author.

## Declarations

### Declaration

All co-authors take full responsibility for the integrity and accuracy of all aspects of the work.
